# Supplementary material for: Modeling glioblastoma heterogeneity as a dynamic network of cell states
Source: Mol Syst Biol. 2021 Sep 16;17(9):e10105. doi: 10.15252/msb.202010105 (PMC8444284; doi:10.15252/msb.202010105)
Supplement: Supplementary file 6 — Source Data for Figure 5 [file MSB-17-e10105-s004.zip › Figure5A_sourcedata/GSEA_3071/nosigdb_stateIV.GseaPreranked.1622795164626/RICHARDS_NOWAKOWSKI_SCIENCE_2017_IN_CTX_CGE2_UPREG.html]

Details for gene set RICHARDS\_NOWAKOWSKI\_SCIENCE\_2017\_IN\_CTX\_CGE2\_UPREG[GSEA]

|  || Dataset | state53071 |
| Phenotype | NoPhenotypeAvailable |
| Upregulated in class | na\_pos |
| GeneSet | RICHARDS\_NOWAKOWSKI\_SCIENCE\_2017\_IN\_CTX\_CGE2\_UPREG |
| Enrichment Score (ES) | 0.515086 |
| Normalized Enrichment Score (NES) | 3.026073 |
| Nominal p-value | 0.0 |
| FDR q-value | 0.0 |
| FWER p-Value | 0.0 |
Table: GSEA Results Summary

  

Fig 1: Enrichment plot: RICHARDS\_NOWAKOWSKI\_SCIENCE\_2017\_IN\_CTX\_CGE2\_UPREG      
 Profile of the Running ES Score & Positions of GeneSet Members on the Rank Ordered List

  

| PROBE | GENE SYMBOL | GENE\_TITLE | RANK IN GENE LIST | RANK METRIC SCORE | RUNNING ES | CORE ENRICHMENT || 1 | DCX |  |  | 4 | 2.752 | 0.0242 | Yes |
| 2 | DLX5 |  |  | 17 | 2.283 | 0.0426 | Yes |
| 3 | LRRN3 |  |  | 29 | 2.015 | 0.0587 | Yes |
| 4 | SLCO5A1 |  |  | 35 | 1.977 | 0.0757 | Yes |
| 5 | CHL1 |  |  | 37 | 1.968 | 0.0934 | Yes |
| 6 | CADM1 |  |  | 38 | 1.965 | 0.1112 | Yes |
| 7 | DLX2 |  |  | 50 | 1.858 | 0.1259 | Yes |
| 8 | DLX1 |  |  | 62 | 1.768 | 0.1398 | Yes |
| 9 | CDH2 |  |  | 68 | 1.735 | 0.1546 | Yes |
| 10 | DCLK2 |  |  | 74 | 1.704 | 0.1691 | Yes |
| 11 | SDK2 |  |  | 85 | 1.618 | 0.1818 | Yes |
| 12 | PDP1 |  |  | 89 | 1.579 | 0.1956 | Yes |
| 13 | TCF4 |  |  | 96 | 1.551 | 0.2085 | Yes |
| 14 | NFIB |  |  | 117 | 1.491 | 0.2181 | Yes |
| 15 | RAB3IP |  |  | 124 | 1.474 | 0.2304 | Yes |
| 16 | PROX1 |  |  | 126 | 1.468 | 0.2435 | Yes |
| 17 | NCAM1 |  |  | 129 | 1.456 | 0.2563 | Yes |
| 18 | RNF180 |  |  | 132 | 1.446 | 0.2690 | Yes |
| 19 | RGS12 |  |  | 133 | 1.439 | 0.2821 | Yes |
| 20 | CCDC88A |  |  | 135 | 1.429 | 0.2949 | Yes |
| 21 | CHD7 |  |  | 144 | 1.386 | 0.3059 | Yes |
| 22 | DLX6 |  |  | 145 | 1.384 | 0.3185 | Yes |
| 23 | TMTC4 |  |  | 147 | 1.382 | 0.3308 | Yes |
| 24 | ZEB2 |  |  | 164 | 1.339 | 0.3398 | Yes |
| 25 | NRXN3 |  |  | 170 | 1.321 | 0.3509 | Yes |
| 26 | PIAS2 |  |  | 187 | 1.285 | 0.3594 | Yes |
| 27 | GAD1 |  |  | 190 | 1.281 | 0.3707 | Yes |
| 28 | ROBO1 |  |  | 193 | 1.273 | 0.3818 | Yes |
| 29 | CXCR4 |  |  | 197 | 1.269 | 0.3927 | Yes |
| 30 | SLAIN1 |  |  | 203 | 1.257 | 0.4032 | Yes |
| 31 | ASAP1 |  |  | 211 | 1.231 | 0.4130 | Yes |
| 32 | CADPS |  |  | 220 | 1.205 | 0.4224 | Yes |
| 33 | SLC5A3 |  |  | 229 | 1.188 | 0.4316 | Yes |
| 34 | GAS2L3 |  |  | 244 | 1.161 | 0.4394 | Yes |
| 35 | GNAQ |  |  | 265 | 1.110 | 0.4456 | Yes |
| 36 | PCDH9 |  |  | 301 | 1.034 | 0.4482 | Yes |
| 37 | MAGI1 |  |  | 355 | 0.969 | 0.4467 | Yes |
| 38 | SOX4 |  |  | 377 | 0.928 | 0.4511 | Yes |
| 39 | WWP1 |  |  | 401 | 0.906 | 0.4548 | Yes |
| 40 | PDZRN3 |  |  | 405 | 0.904 | 0.4625 | Yes |
| 41 | ARX |  |  | 415 | 0.891 | 0.4688 | Yes |
| 42 | ELOVL6 |  |  | 437 | 0.867 | 0.4726 | Yes |
| 43 | INTU |  |  | 463 | 0.846 | 0.4754 | Yes |
| 44 | RTN1 |  |  | 471 | 0.836 | 0.4816 | Yes |
| 45 | APC |  |  | 492 | 0.822 | 0.4852 | Yes |
| 46 | ZNF704 |  |  | 498 | 0.818 | 0.4917 | Yes |
| 47 | CD24 |  |  | 545 | 0.770 | 0.4897 | Yes |
| 48 | TMOD2 |  |  | 591 | 0.732 | 0.4877 | Yes |
| 49 | AP1S2 |  |  | 608 | 0.723 | 0.4911 | Yes |
| 50 | BRD3 |  |  | 626 | 0.710 | 0.4943 | Yes |
| 51 | PAK2 |  |  | 631 | 0.707 | 0.4999 | Yes |
| 52 | SCG3 |  |  | 632 | 0.707 | 0.5063 | Yes |
| 53 | PAIP2 |  |  | 658 | 0.683 | 0.5077 | Yes |
| 54 | ARID1B |  |  | 686 | 0.667 | 0.5085 | Yes |
| 55 | MALAT1 |  |  | 739 | 0.638 | 0.5042 | Yes |
| 56 | MIDN |  |  | 795 | 0.604 | 0.4990 | Yes |
| 57 | ASAH1 |  |  | 833 | 0.587 | 0.4972 | Yes |
| 58 | UBXN7 |  |  | 891 | 0.564 | 0.4912 | Yes |
| 59 | BAG5 |  |  | 917 | 0.554 | 0.4914 | Yes |
| 60 | SLC6A1 |  |  | 927 | 0.549 | 0.4947 | Yes |
| 61 | NDUFB4 |  |  | 999 | 0.522 | 0.4856 | Yes |
| 62 | GNG2 |  |  | 1019 | 0.515 | 0.4866 | Yes |
| 63 | SETD2 |  |  | 1021 | 0.514 | 0.4911 | Yes |
| 64 | RAB11B |  |  | 1024 | 0.513 | 0.4953 | Yes |
| 65 | LUC7L |  |  | 1037 | 0.509 | 0.4976 | Yes |
| 66 | PAK3 |  |  | 1046 | 0.506 | 0.5007 | Yes |
| 67 | CGGBP1 |  |  | 1062 | 0.503 | 0.5023 | Yes |
| 68 | NPAS3 |  |  | 1068 | 0.502 | 0.5059 | Yes |
| 69 | QKI |  |  | 1078 | 0.500 | 0.5087 | Yes |
| 70 | UQCR11 |  |  | 1114 | 0.488 | 0.5063 | Yes |
| 71 | BTG1 |  |  | 1123 | 0.486 | 0.5092 | Yes |
| 72 | GNPTAB |  |  | 1127 | 0.486 | 0.5130 | Yes |
| 73 | IGF1R |  |  | 1140 | 0.482 | 0.5151 | Yes |
| 74 | DCUN1D1 |  |  | 1202 | 0.464 | 0.5075 | No |
| 75 | PRKCA |  |  | 1214 | 0.461 | 0.5095 | No |
| 76 | SOGA1 |  |  | 1242 | 0.453 | 0.5084 | No |
| 77 | KANSL1 |  |  | 1345 | 0.429 | 0.4925 | No |
| 78 | STT3B |  |  | 1394 | 0.417 | 0.4870 | No |
| 79 | FAM110B |  |  | 1440 | 0.405 | 0.4819 | No |
| 80 | ZNF302 |  |  | 1520 | 0.385 | 0.4701 | No |
| 81 | PPP2CB |  |  | 1580 | 0.370 | 0.4620 | No |
| 82 | AKAP6 |  |  | 1627 | 0.363 | 0.4564 | No |
| 83 | ENSA |  |  | 1661 | 0.357 | 0.4532 | No |
| 84 | MTSS1 |  |  | 1708 | 0.348 | 0.4475 | No |
| 85 | KCNJ2 |  |  | 1722 | 0.346 | 0.4481 | No |
| 86 | TBC1D23 |  |  | 1753 | 0.339 | 0.4453 | No |
| 87 | WDR83OS |  |  | 1761 | 0.337 | 0.4470 | No |
| 88 | EP300 |  |  | 1816 | 0.328 | 0.4395 | No |
| 89 | ERBB4 |  |  | 1843 | 0.325 | 0.4374 | No |
| 90 | BOD1L1 |  |  | 1849 | 0.324 | 0.4394 | No |
| 91 | RAP2A |  |  | 1903 | 0.314 | 0.4320 | No |
| 92 | LRCH2 |  |  | 1962 | 0.304 | 0.4235 | No |
| 93 | SHISA9 |  |  | 2019 | 0.293 | 0.4153 | No |
| 94 | MED28 |  |  | 2049 | 0.290 | 0.4123 | No |
| 95 | SF3B1 |  |  | 2265 | 0.260 | 0.3730 | No |
| 96 | RBM25 |  |  | 2275 | 0.260 | 0.3736 | No |
| 97 | MCFD2 |  |  | 2343 | -0.251 | 0.3628 | No |
| 98 | NAPG |  |  | 2346 | -0.252 | 0.3647 | No |
| 99 | SCP2 |  |  | 2368 | -0.255 | 0.3630 | No |
| 100 | KIAA0232 |  |  | 2529 | -0.272 | 0.3344 | No |
| 101 | PTPRD |  |  | 2553 | -0.275 | 0.3325 | No |
| 102 | GTF3C6 |  |  | 2635 | -0.287 | 0.3194 | No |
| 103 | PRRC2C |  |  | 2801 | -0.306 | 0.2901 | No |
| 104 | GRIK2 |  |  | 2858 | -0.314 | 0.2821 | No |
| 105 | FRY |  |  | 2899 | -0.319 | 0.2773 | No |
| 106 | SMC1A |  |  | 2968 | -0.328 | 0.2670 | No |
| 107 | KITLG |  |  | 3197 | -0.360 | 0.2261 | No |
| 108 | ANK3 |  |  | 3216 | -0.363 | 0.2259 | No |
| 109 | FAM204A |  |  | 3314 | -0.377 | 0.2105 | No |
| 110 | IGDCC4 |  |  | 3373 | -0.387 | 0.2028 | No |
| 111 | KPNA3 |  |  | 3423 | -0.394 | 0.1968 | No |
| 112 | SLIRP |  |  | 3464 | -0.402 | 0.1927 | No |
| 113 | DRG1 |  |  | 3610 | -0.430 | 0.1685 | No |
| 114 | SNRPA1 |  |  | 3638 | -0.434 | 0.1672 | No |
| 115 | VDAC1 |  |  | 3662 | -0.437 | 0.1667 | No |
| 116 | WASF2 |  |  | 3818 | -0.469 | 0.1409 | No |
| 117 | CCT3 |  |  | 3930 | -0.494 | 0.1239 | No |
| 118 | TOMM5 |  |  | 3941 | -0.496 | 0.1264 | No |
| 119 | SSRP1 |  |  | 4112 | -0.544 | 0.0984 | No |
| 120 | RIMS1 |  |  | 4201 | -0.568 | 0.0865 | No |
| 121 | HSPA9 |  |  | 4410 | -0.639 | 0.0519 | No |
| 122 | EIF3J |  |  | 4426 | -0.643 | 0.0549 | No |
| 123 | ITGB1BP1 |  |  | 4445 | -0.653 | 0.0573 | No |
| 124 | VCAN |  |  | 4464 | -0.661 | 0.0598 | No |
| 125 | NOP58 |  |  | 4539 | -0.699 | 0.0518 | No |
| 126 | SYT1 |  |  | 4552 | -0.707 | 0.0559 | No |
| 127 | MIAT |  |  | 4622 | -0.748 | 0.0493 | No |
| 128 | PSMA3 |  |  | 4667 | -0.773 | 0.0477 | No |
| 129 | EIF5A |  |  | 4738 | -0.821 | 0.0416 | No |
| 130 | CALB2 |  |  | 4770 | -0.840 | 0.0432 | No |
| 131 | NR2F2 |  |  | 4870 | -0.937 | 0.0325 | No |
| 132 | ATAD5 |  |  | 4934 | -0.998 | 0.0294 | No |
| 133 | NR2F1 |  |  | 5135 | -1.386 | 0.0031 | No |
| 134 | ARL4C |  |  | 5141 | -1.392 | 0.0148 | No |
| 135 | CAMK2N1 |  |  | 5174 | -1.531 | 0.0225 | No |
Table: GSEA details [plain text format]

  

Fig 2: RICHARDS\_NOWAKOWSKI\_SCIENCE\_2017\_IN\_CTX\_CGE2\_UPREG: Random ES distribution      
 Gene set null distribution of ES for **RICHARDS\_NOWAKOWSKI\_SCIENCE\_2017\_IN\_CTX\_CGE2\_UPREG**
